# Enteral Nutrition Related Complications Relevant to Alteration of Formulas in Two Critically Ill Pediatric Patients

**DOI:** 10.4021/gr568w

**Published:** 2013-09-09

**Authors:** Nobuhiro Akuzawa, Aya Murata Takeuchi, Jun Tsukagoshi, Ryoko Kaneko, Hiroshi Naito, Takahisa Mizuno, Yasuo Sunaga, Masahiko Tashiro

**Affiliations:** a Nutrition Support Team, Social Insurance Gunma Chuo General Hospital, 1-7-13 Koun-cho, Maebashi, Gunma 371-0025, Japan; b Department of Pediatrics, Social Insurance Gunma Chuo General Hospital, 1-7-13 Koun-cho, Maebashi, Gunma 371-0025, Japan

**Keywords:** Enteral feeding, Pediatric, Adverse event, Formula change

## Abstract

The early institution of enteral nutrition is associated with beneficial outcomes and intestinal growth in pediatric patients. However, the number, frequency, and types of unfavorable events occurring with particular formulas are undefined. We experienced unexpected complications in two cases following a change in formula. One case diagnosed with myotubular myopathy experienced highly-increased gastric residuals and watery diarrhea leading to decreased calorie intake and weight loss. The second case with campomelic dysplasia suffered liver dysfunction and fever. In both cases, symptoms developed soon after of the change in formula and improved after resumption of the previous formula. Both cases had undergone tracheostomy and artificial ventilation, and had a history of feeding the same formula for an extended period of time. In chronic care patients such as ours, a change in formula may cause unexpected adverse events; therefore, caution is warranted.

## Introduction

Enteral nutrition (EN) is considered a favorable means of nutrient intake in critically ill adult patients with a functional gastrointestinal system because of its lower cost and complication rates compared with parenteral nutrition [1]. Early institution of EN is associated with beneficial outcomes in both adult and pediatric patients [2]. Gastrointestinal priming prior to full EN accelerates increasing serum gastrin levels during the first weeks of life in very low birth weight infants, suggesting a contribution to intestinal growth [3].

Diarrhea, abdominal distension, vomiting, and high gastric residuals are reported complications of enteral feeding [4]. Increased gastric residuals may be a cause of bronchoaspiration leading to secondary pneumonia [5]. However, the number, frequency, and types of unfavorable events occurring during the use of particular formulas remain undefined.

We present two pediatric cases that experienced unexpected complications related to an unavoidable change in formula because of product shortages following the Japanese earthquake in March, 2011. One case developed high gastric residuals and diarrhea, and the other case experienced progressive liver dysfunction. Symptoms appeared during the use of substitute formulas and completely resolved after resumption of the previous formula.

## Case Report

### Case 1

A 2-year-6-month-old male child had been admitted to a pediatric intensive care unit since birth. He was born floppy, requiring resuscitation at a gestational age of 37 weeks. His birth weight was 2,260 g, his five-minute Apgar score was 5, and shortly after his birth he was intubated, ventilated, and transferred to a neonatal intensive care unit. At 1 year of age, he underwent tracheostomy for long-term mechanical ventilation and muscle biopsy was performed, confirming a diagnosis of myotubular myopathy. An older brother had also been diagnosed with myotubular myopathy. He had no other congenital skeletal, cardiovascular, or gastrointestinal system deformities.

Enteral nutrition using mother’s milk was begun on day 3 after birth via nasogastric tube. At 6 months of age, mixed feeding of mother’s milk with synthetic milk was introduced and was completely replaced with synthetic milk one month later. His 7-month-old body weight and daily calorie intake were 4,100 g and 500 kcal/day, respectively. His body weight at 1 year was only 4,900 g and his height was 70 cm. Ensure Liquid (Abbott Nutrition, Columbus, OH, USA), containing more calories than synthetic milk, was introduced successfully soon after his first birthday, increasing the daily calorie intake to 600 kcal/day. However, attempts to increase the intake or to change to a high-density liquid diet failed because of increased gastric residuals. Because of this, his daily calorie intake remained at 600 kcal/day.

When the earthquake occurred on March 11, 2011, the patient’s body height and weight were 83 cm and 6,380 g, respectively; he was receiving furosemide (5 mg/day) and spironolactone (5 mg/day); and was fed 6 times a day (100 kcal/100 mL + 20 mL water × 6). Four weeks after the earthquake, the stock of Ensure Liquid in our hospital ran low and an alternate formula was necessary. This was due to a widely disordered distribution system in East Japan. We chose MEIN (Meiji Co., Ltd, Tokyo, Japan) because of the similar composition of carbohydrates, protein and fat balance, and equal calorie content (1 kcal/mL).

**Figure 1:**
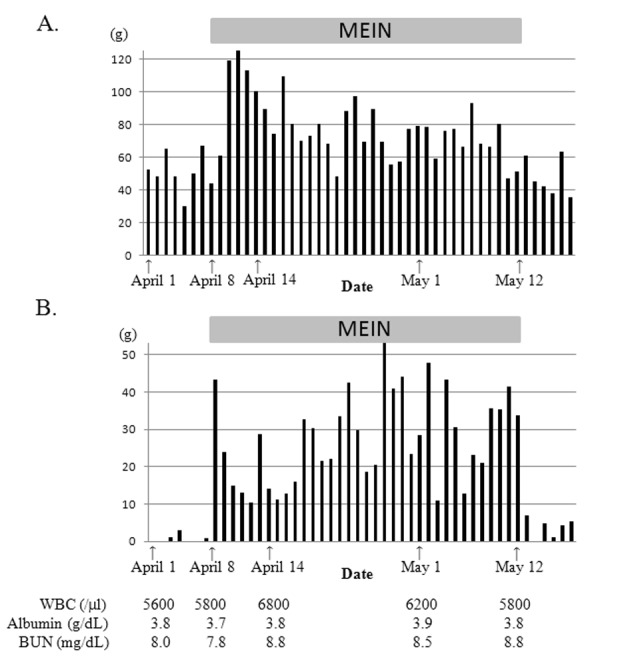
Daily excreted feces amount (A) and total gastric residuals (B) of case 1. Gastric residuals increased markedly and watery diarrhea occurred after introducing MEIN. After reducing MEIN from 600 mL/day to 550 mL/day, the watery diarrhea improved. However, increased gastric residuals persisted until we discontinued MEIN and decreased dramatically after reintroducing ensure liquid.

Soon after the introduction of MEIN, the amount of excreted feces nearly doubled, became watery, and the gastric residuals increased (Fig. 1). However, pre- and post-prandial plasma glucose levels of this patient remained normal (70 - 90 mg/dL and 90 - 120 mg/dL, respectively), and any other symptoms suggesting dumping syndrome were not observed. Additionally, serum zinc level was also normal. We reduced the amount of MEIN from 600 mL/day to 550 mL/day when these symptoms did not improve. Despite additional oral rehydration solution (50 mL/day), the patient’s body weight decreased to 6,170 g 3 weeks after the formula change. Watery diarrhea gradually improved, but increased gastric residuals continued until the discontinuation of MEIN, 11 days later. Gastric residuals dramatically decreased soon after feeding with Ensure Liquid resumed. The patient gained weight and reached 6,910 g approximately 6 weeks after Ensure feeding was reintroduced. Serum albumin level was approximately 3.5 g/dL with no data suggesting severe dehydration or malnutrition.

### Case 2

A 3-year-old female child had been admitted to a pediatric intensive care unit since birth. She was born at a gestational age of 39 weeks with a birth weight of 2,576 g, bilateral bowing and shortening of the long bones, clubfeet, dislocated hips, underdeveloped shoulder blades, and brevicollis. Her five-minute Apgar score was 9, but oxygen saturation often decreased below 90% with nasal breathing. Based on the skeletal characteristics, we diagnosed her as having campomelic dysplasia, although conventional cytogenetics showed normal karyotype (46, XX) and a high-resolution banding study of chromosome 17 showed no abnormalities. Further investigation revealed weakened cartilage forming the upper respiratory tract and when she was 48 days old, she was intubated for artificial respiration because of respiratory failure resulting from bronchomalacia. At the age of 2, she underwent tracheostomy and required a respirator.

Enteral nutrition using mother’s milk was started on day 2 after birth via nasogastric tube. At 2 months of age, synthetic formula was introduced and mixed with mother’s milk, completely replacing mother’s milk at 4 months of age. Her body weight at 1 year of age was 3,100 g, which is when Ensure Liquid replaced synthetic milk. At 2 years of age, her body weight was only 3,300 g, but each time we attempted to increase her calorie intake by feeding more calorie-dense liquid meals or increasing the volume of Ensure Liquid, increased gastric residuals or vomiting occurred. When the earthquake occurred, her height and weight were 55 cm and 3,350 g, respectively; she was receiving furosemide (8 mg/day) and spironolactone (8 mg/day); and was being fed Ensure Liquid 5 times a day (60 mL + 15 mL water × 5). As in case 1, we were forced to choose a substitute formula. Approximately 6 weeks after the earthquake, we began feeding Terumeal-mini (Terumo Corporation, Tokyo, Japan), which contained more calories than Ensure Liquid (1.6 kcal/mL), 5 times a day (40 mL + 40 mL water × 5).

**Figure 2:**
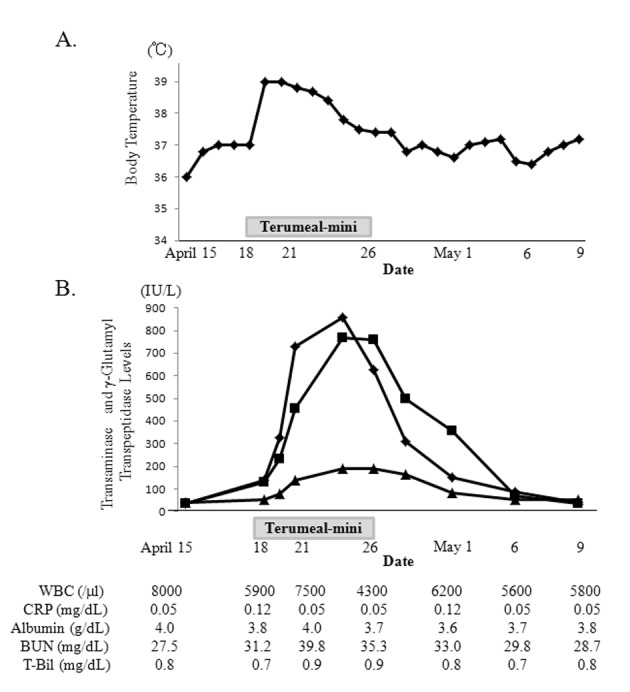
Changes in body temperature (A) and aspartate aminotransferase (AST; rectangle), alanine transaminase (ALT; square) and *γ-glutamyl transpeptidase (γ-GTP; triangle)* levels (B) in case 2. After beginning Terumeal-mini, the patient became feverish and liver dysfunction deteriorated rapidly. AST and ALT peaked at 857 IU/L and 768 IU/L, respectively. *γ-GTP* peaked at 192 IU/L. Total bilirubin level remained normal and blood urea nitrogen level temporarily increased when the patient had a fever. After discontinuing the Terumeal-mini and reintroducing the Ensure Liquid, the fever resolved and the liver dysfunction normalized.

Two days after starting Terumeal-mini, the patient became feverish and liver function deteriorated rapidly (Fig. 2). However, the complete and differential white blood cell counts, C-reactive protein and total bilirubin levels remained normal. Blood urea nitrogen level temporarily increased when the patient had a fever. Blood ammonia and lactic acid levels were normal. Abdominal ultrasonography and chest X-ray showed no abnormalities. Sputum, urine, feces, and blood culture samples detected no pathogens and a rapid antigen test for influenza, adenovirus, and pneumococcus was negative. Hepatitis B surface antigen, antibodies for hepatitis A virus, hepatitis C virus, cytomegalovirus and herpes simplex virus, and IgM antibody to Epstein-Barr viral capsid antigen, were negative. Her fever gradually and spontaneously improved without treatment but the liver dysfunction persisted. We suspected that the liver dysfunction was related to the formula change. We acquired and resumed Ensure Liquid 7 days after the fever appeared and the patient’s liver dysfunction gradually normalized over 2 weeks without treatment.

## Discussion

We presented two pediatric cases with complications related to changing the EN formula. Case 1 developed watery diarrhea and increased gastric residuals and case 2 developed fever and liver dysfunction. Problems resolved in both cases after resumption of the previous formula. Both cases underwent tracheostomy and required artificial respiration and both had a history of gastric feeding with the same formula (Ensure Liquid) via nasogastric tube for an extended period of time; one and a half years in case 1 and two years in case 2.

In case 1, increased gastric residuals occurred soon after the introduction of MEIN and was followed by watery diarrhea. Although the diarrhea improved slightly by reduced administration of MEIN and resolved spontaneously in 10 days, the amount of the increased gastric residuals remained unchanged. As shown in Table 1, both MEIN and Ensure Liquid have the same caloric content and a similar ratio of fat, protein and carbohydrates. However, the osmolality of MEIN is higher than Ensure Liquid because it contains both low-molecular-weight protein hydrolysates and disaccharide (isomaltulose) instead of polysaccharide (dextrin) and sucrose, which are more commonly used. The sources of fat and protein are also quite different between these two products. High gastric residuals are the most common reason for decreasing or discontinuing feedings in preterm infants [6]. Also, abdominal distension and apnea/bradycardia are frequent signs of feeding intolerance (FI) in preterm infants [6]. Changing the formula may affect FI and the incidence of FI is more frequently reported in preterm infants fed liquid formula vs. powdered formula [7]. However, comparison reports of each formula’s effects on FI are rare. Pascale et al [8] investigated relationships between gastric function and various formulas in low birth weight infants and revealed that formulas having a high osmolality showed greater gastric retention. However, the fatty acid chain length of the triglyceride in the formula did not affect gastric emptying [8]. The osmolality of commonly-used pediatric formulas ranges from 260 - 600 mOsm/kg [9], and the osmolality of MEIN (600 mOsm/kg) is comparable to other formulas. However, in case 1, the high osmolality may have had a major impact on increased gastric residuals, leading to decreased calorie intake and weight loss.

**Table 1:** Comparison of Ensure Liquid, MEIN and Terumeal-Mini

	Ensure Liquid	MEIN	Terumeal-mini
Caloric Content (kcal/mL)	1.0	1.0	1.6
Free Water Content (g/mL)	0.85	0.84	0.47
Fiber Content (g/mL)	0	0.012	0.005
Osmotic Pressure (mOsm/kg)	330	600	470
Calorie-based Combination Ratio of Fat, Protein and Carbohydrates	1:1:4	1:1.8:3.8	1:1:3.2
Protein Source	Casein, soy	Casein, whey	Casein
Fat Source	Corn, soy	Canola, palm, fish, MCT	Soy
Carbohydrates	Sucrose	Isomaltulose, dextrin	Sucrose, dextrin

MCT: medium-chain triglycerides.

In case 2, the cause of the fever and liver dysfunction required investigation. As shown in Table 1, caloric content and osmolality of Terumeal-mini are approximately 1.5 times that of Ensure Liquid. In children, refeeding syndrome may develop without clinical signs, detectable only through laboratory findings [10]. The total calorie intake of Terumeal-mini at 320 kcal/day was not significantly greater in this patient than the previous calorie intake of 300 kcal/day. Also, the normal serum potassium, phosphate, and magnesium levels did not indicate refeeding syndrome. Patients with campomelic dysplasia can have liver cysts or enlarged liver [11, 12], but these were not seen in this patient. Results of screening tests for congenital metabolic diseases at birth were also normal and she had thrived without any nutrient limitation, suggesting that causes other than metabolic diseases may have contributed to her fever and liver dysfunction, such as food allergies. Liver dysfunction due to cow’s milk protein allergy has been reported previously in Japan [13-15]. However, the reason for liver dysfunction and fever seen in case 2 cannot be explained by cow’s milk allergy, because both Ensure Liquid and Terumeal-mini contain cow’s milk-derived protein. Also, unlike cases of cow’s milk allergy, eosinophilia was not observed in case 2. It is remotely possible that allergenic denatured proteins or food additives specific to Terumeal-mini may have been responsible for the fever and liver dysfunction. Only gellan gum and polysaccharides obtained from fermented starch were included in Terumeal-mini, to our knowledge, but no cases showing similar symptoms or findings after gellan gum administration have been reported, and therefore, the causative agents remain unclear.

In conclusion, we experienced two pediatric cases with adverse effects following a change in EN formulas. One experienced persistent increased gastric residuals leading to decreased calorie intake and weight loss, and the other case experienced fever and liver dysfunction. Both cases underwent tracheostomy and artificial ventilation, and had a history of gastric feeding with the same formula for an extended period of time. Both cases had extremely low body weight for their age. In chronic-care cases such as ours, changing EN formulas may cause unexpected adverse events and therefore, caution should be exercised.

## References

[1] Heyland DK, Dhaliwal R, Drover JW, Gramlich L, Dodek P (2003). Canadian clinical practice guidelines for nutrition support in mechanically ventilated, critically ill adult patients. JPEN J Parenter Enteral Nutr.

[2] Chellis MJ, Sanders SV, Webster H, Dean JM, Jackson D (1996). Early enteral feeding in the pediatric intensive care unit. JPEN J Parenter Enteral Nutr.

[3] Meetze WH, Valentine C, McGuigan JE, Conlon M, Sacks N, Neu J (1992). Gastrointestinal priming prior to full enteral nutrition in very low birth weight infants. J Pediatr Gastroenterol Nutr.

[4] Elpern EH, Stutz L, Peterson S, Gurka DP, Skipper A (2004). Outcomes associated with enteral tube feedings in a medical intensive care unit. Am J Crit Care.

[5] Montejo JC, Grau T, Acosta J, Ruiz-Santana S, Planas M, Garcia-De-Lorenzo A, Mesejo A (2002). Multicenter, prospective, randomized, single-blind study comparing the efficacy and gastrointestinal complications of early jejunal feeding with early gastric feeding in critically ill patients. Crit Care Med.

[6] Lucchini R, Bizzarri B, Giampietro S, De Curtis M (2011). Feeding intolerance in preterm infants. How to understand the warning signs. J Matern Fetal Neonatal Med.

[7] Surmeli-Onay O, Korkmaz A, Yigit S, Yurdakok M (2013). Feeding intolerance in preterm infants fed with powdered or liquid formula: a randomized controlled, double-blind, pilot study. Eur J Pediatr.

[8] Pascale JA, Mims LC, Greenberg MG, Alexander JB (1978). Gastric response in low birth weight infants fed various formulas. Biol Neonate.

[9] Joeckel RJ, Phillips SK (2009). Overview of infant and pediatric formulas. Nutr Clin Pract.

[10] Lenicek Krleza J, Misak Z, Jadresin O, Skaric I (2013). Refeeding syndrome in children with different clinical aetiology. Eur J Clin Nutr.

[11] Massardier J, Roth P, Michel-Calemard L, Rudigoz RC, Bouvier R, Dijoud F, Arnould P (2008). Campomelic dysplasia: echographic suspicion in the first trimester of pregnancy and final diagnosis of two cases. Fetal Diagn Ther.

[12] Houston CS, Opitz JM, Spranger JW, Macpherson RI, Reed MH, Gilbert EF, Herrmann J (1983). The campomelic syndrome: review, report of 17 cases, and follow-up on the currently 17-year-old boy first reported by Maroteaux et al in 1971. Am J Med Genet.

[13] Saito M, Obi M, Kimura M (2005). Infantile hepatic dysfunction improved by elimination of cows' milk formulas. Pediatr Allergy Immunol.

[14] Yada K, Yoshida K, Sakurai Y, Kimura M, Yasuhara H, Tanaka I, Yoshioka A (2008). Casein hydrolysate formula-induced liver dysfunction in a neonate with non-immunoglobulin E-mediated cow's milk allergy. J Investig Allergol Clin Immunol.

[15] Matsumoto K, Esumi G, Teshiba R, Nagata K, Hayashida M, Nakatsuji T, Takahashi Y (2008). Cow's milk allergy in extremely short bowel syndrome: report of two infants. E Spen Eur E J Clin Nutr Metab.

